# Acute Retinal Necrosis Associated with Epstein–Barr Virus Successfully Treated with Antiviral Treatment: A Case Report

**DOI:** 10.3390/microorganisms12102065

**Published:** 2024-10-15

**Authors:** Heejeong You, Joonhyung Kim

**Affiliations:** Department of Ophthalmology, CHA Bundang Medical Center, CHA University School of Medicine, Seongnam 13496, Republic of Korea

**Keywords:** acute retinal necrosis, Epstein–Barr virus, acyclovir, case report

## Abstract

Epstein–Barr virus (EBV) is a rare cause of acute retinal necrosis (ARN) and is known for its poor prognosis and limited response to conventional antiviral treatment. Herein, we report a case of EBV ARN successfully treated with conventional systemic acyclovir and intravitreal ganciclovir injection. An 85-year-old man presented with visual disturbance of the right eye from 10 days prior. His visual acuity was 20/200 in the right eye and slit lamp examination showed keratic precipitates, 4+ anterior chamber cells, and 1+ anterior vitreous cells. Fundus examination revealed multiple retinal hemorrhages and yellow-whitish necrotic lesion. The patient was clinically diagnosed with ARN. A few days later, EBV DNA was identified in the aqueous humor and in the serum PCR assay. The patient received 350 mg of intravenous acyclovir three times a day with oral prednisolone, and an intravitreal ganciclovir injection (2 mg per dose) was given five times. Over the course of seven weeks, systemic acyclovir was switched to 1g of per-oral valaciclovir three times a day, and oral steroids were successfully tapered. His visual acuity improved to 20/100, and the previous necrotic lesion was markedly decreased in size. Intravenous acyclovir combined with intravitreal ganciclovir may yield successful treatment outcomes in acute retinal necrosis caused by EBV.

## 1. Introduction

Acute retinal necrosis (ARN) is a vision-threatening disease characterized by panuveitis and necrosis of the retina. It is an uncommon disease, with approximately 0.63 cases per million people per year [1]. The diagnosis of ARN is primarily based on clinical findings and the identification of a causative virus through antiviral antibody testing and polymerase chain reaction (PCR) [2]. Varicella zoster virus (VZV) is known to be the most common causative agent, followed by herpes simplex virus (HSV) 1 and 2, and other rare causes, such as cytomegalovirus (CMV), Epstein–Barr virus (EBV), and human herpesvirus-6 (HHV-6) have also been reported [2,3,4].

Epstein–Barr virus is a double-stranded DNA virus, well known for opportunistic infections in immunocompromised patients. There were only a few reports regarding EBV-associated ARN until now, which showed poor visual prognoses [5]. In this report, we present a rare case of EBV ARN in an immunocompetent patient who showed a good response to conventional treatment.

Written informed consent was obtained from the patient.

## 2. Case Report

An 85-year-old male patient presented to our hospital with visual disturbance of the right eye from ten days prior. Visual acuity was 20/200 in the right eye and 20/25 in the left eye. The patient was receiving medication for hypertension, diabetes mellitus, myocardial infarction with stent insertion, stage 4 chronic kidney disease, and cerebral atherosclerosis. He was not on any steroids or immunosuppressive agents. He was followed up with for diabetic retinopathy at a primary ophthalmology clinic.

**Figure 1:**
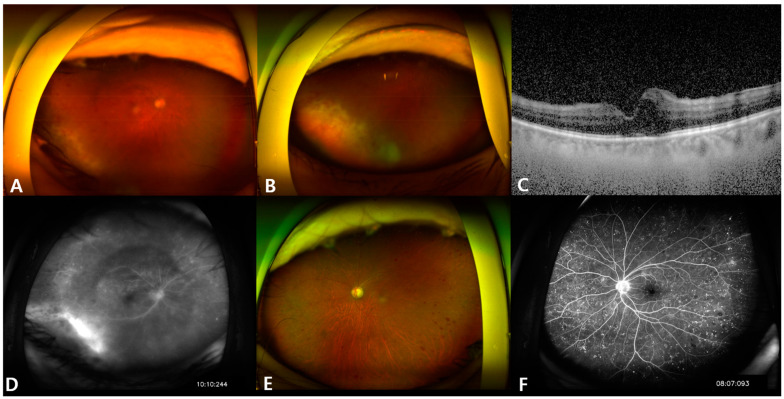
(**A**,**B**) Wide fundus photography of the right eye. The view is hazy due to anterior chamber cells and vitreous opacity. Multiple retinal hemorrhages and a yellow-whitish necrotic lesion are noted at the inferotemporal area. (**C**) Mild macular edema of the right eye. (**D**) Dye leakage is noted at the site corresponding to the necrotic lesion. (**E**) Wide fundus photography of the left eye. Multiple retinal hemorrhages are noted, but there are no necrotic lesions. (**F**) Microaneurysms due to diabetic retinopathy.

Slit lamp examination revealed cornea endothelial precipitates and 4+ cells in the anterior chamber, and 1+ cells in the anterior vitreous of the right eye. The anterior segment of the left eye was normal. Dilated fundus examination revealed multiple retinal hemorrhages and a yellow-whitish necrotic lesion at the inferotemporal area of the right eye (Figure 1A,B), and optical coherence tomography (OCT) revealed macular edema (Figure 1C). Fluorescein angiography (FAG) demonstrated dye leakage indicating vasculitis of the right eye (Figure 1D). The left eye showed no specific signs during the slit lamp examination. Fundus examination showed multiple peripheral retinal hemorrhages, presumably due to known diabetic retinopathy (Figure 1E). Consistent with the fundus findings, FAG revealed multiple microaneurysms without any dye leakage in the left eye (Figure 1F).

The patient was clinically diagnosed with acute retinal necrosis of the right eye. The administration of moxifloxacin eyedrops and 1% prednisolone acetate eyedrops was initiated. Doses of 350 mg of intravenous acyclovir three times a day and 60 mg of oral prednisolone were administered. A 360-degree barrier laser retinopexy was performed on the right eye, and panretinal photocoagulation was performed on the left eye.

The laboratory results revealed a normal range of white blood cell counts. VZV, HSV, CMV, and Toxoplasma all tested positive for IgG but negative for IgM. The EBV early antigen IgG and viral capsid antigen IgG were positive, and the EBV DNA PCR assay was also positive. Additionally, EBV DNA was detected in the patient’s aqueous humor PCR assay, whereas VZV, HSV, and CMV were not detected.

**Figure 2:**
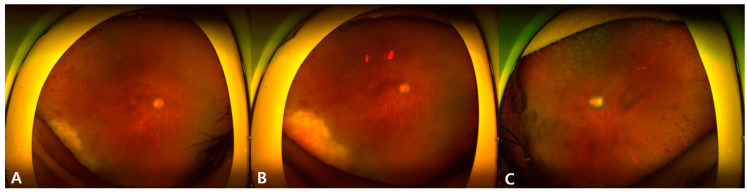
Wide fundus photography of the right eye (**A**,**B**) and the left eye (**C**) after ten days of intravenous antiviral treatment. Note the decrease in the extent of the necrotic lesion of the right eye, especially in the 6 o’clock direction. Medial opacity in (**C**) is due to corneal abrasion caused by panretinal photocoagulation.

Based on the laboratory results, EBV was suspected to be the causative agent of acute retinal necrosis. An intravitreal ganciclovir injection (2 mg per dose) was administered four times in the span of three days. After 10 days of treatment, a slight decrease in the size of the previous necrotic lesion of the right eye was noted (Figure 2A,B). A minimal increase in retinal hemorrhage in the left eye was noted, but there was no necrotic lesion (Figure 2C).

Intravenous acyclovir was changed to 1g of oral valaciclovir three times a day, and oral prednisolone was tapered to 50 mg per day. The patient did not show any progression for three days after changing to oral antivirals, so he was discharged and followed up with at the outpatient clinic.

One week after discharge, a fifth intravitreal ganciclovir injection was administered. Visual acuity was 20/200 in the right eye and 20/40 in the left eye. The anterior chamber and vitreous inflammation were stationary with rare cells, and the retinal necrotic lesion was decreasing. Oral prednisolone was further tapered, but the same dose of valaciclovir was continued.

**Figure 3:**
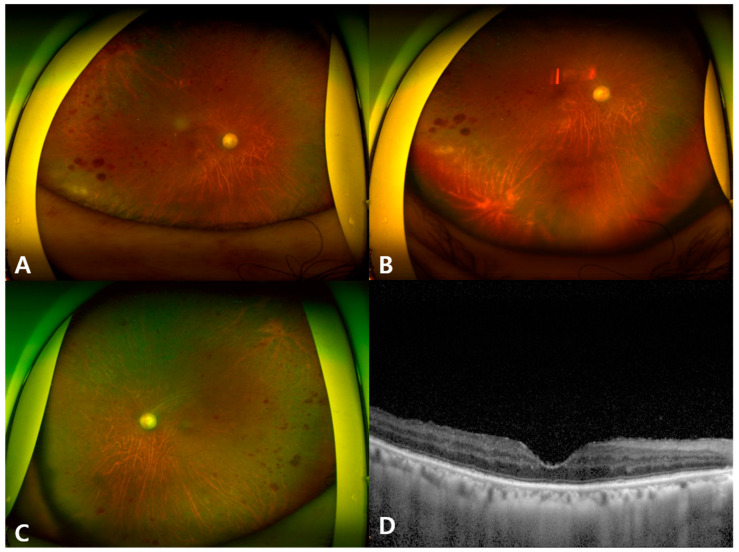
Wide fundus photography of the right eye (**A**,**B**) and the left eye (**C**) 41 days after initial presentation. Macular OCT of the right eye (**D**).

Forty-one days after initial presentation, the visual acuity was 20/100 in the right eye and 20/40 in the left eye. The cornea and anterior chamber were clear in both eyes. Vitreous opacity was decreased but remained in the right eye, and the size of the previous necrotic lesion was much decreased (Figure 3A,B). The left eye did not show any specific signs except the known retinal hemorrhage (Figure 3C). No macular edema was noted using OCT (Figure 3D).

## 3. Discussion and Conclusions

ARN is a clinical diagnosis, and the panuveitis and retinal findings for this patient were consistent with the disease. In addition, considering the patient’s aqueous humor PCR results and hematologic results, it is reasonable to consider EBV as the causative pathogen. Nevertheless, prompt diagnosis and treatment are very important in ARN, and even if the identity of the viral pathogen is not clear, it is important to start antiviral treatment immediately rather than waiting for laboratory results.

There are few previous reports in which EBV was detected with VZV, but several cases reported EBV as the sole pathogen [5,6,7,8,9,10,11,12,13,14]. The majority of these cases resulted in poor visual outcomes, and some patients underwent vitrectomy and even enucleation. Compared with previous cases, our case showed a relatively good prognosis with conventional antiviral treatment.

EBV is a highly prevalent virus worldwide, affecting more than 90% of the population [15]. While the majority of the population does not develop fatal symptoms, it can cause opportunistic infections in immunocompromised patients or undergo a reactivation period resulting in lymphoproliferative disorders. More than half of the previously reported EBV ARN cases involved immunocompromised patients [5]. Despite there being several conditions of note in his medical history, our patient was an immunocompetent patient who did not receive any steroids or immunosuppressants.

Some reports have highlighted the importance of quantitative PCR in detecting EBV. This point is very valid, especially in light of the high seropositive rate of EBV. However, the cost-effectiveness of performing quantitative PCR should be considered, given the low prevalence of EBV in patients with ARN. Additionally, given that the prompt administration of antiviral drugs is a top priority in ARN, the effect of antiviral drugs should be considered when quantitative blood tests are performed after confirming qualitative PCR results.

We performed prophylactic laser retinopexy to prevent total retinal detachment. Due to vitreous opacity, the laser had to be used several times. Prophylactic lasers and early vitrectomy are being discussed as adjunctive treatments, but there are no large studies on this topic as of yet [16].

Systemic antiviral treatment is the standard treatment for ARN. However, adjuvant intravitreal antiviral treatment has been widely used in recent years [16,17]. Two comparative studies have assessed the effect of intravitreal foscarnet combined with systemic antivirals. Both studies reported favorable results for the combination treatment group, with a reduction in the incidence of retinal detachment or poor visual outcome [18,19].

Due to its rarity, there are no standard guidelines for treating EBV-related ARN. Additionally, previous studies have reported poor visual outcomes using systemic acyclovir or ganciclovir. Recent studies have reported on the use of alternative treatments in patients who respond poorly to conventional treatment using systemic acyclovir. Suzuki et al. reported successful treatment using systemic foscarnet in acyclovir-resistant EBV ARN [5]. Also, intravitreal methotrexate injection showed successful results in EBV necrotizing retinitis [8,14].

However, this patient showed favorable outcomes with conventional systemic antivirals and intravitreal ganciclovir injection. Therefore, when EBV ARN is diagnosed, the authors recommend initially applying conventional therapy, then considering other treatment options based on the response.

## Data Availability

The data that support the findings of this case report are available from the corresponding author upon reasonable request.
